# Editorial: Machine Learning Used in Biomedical Computing and Intelligence Healthcare, Volume I

**DOI:** 10.3389/fgene.2021.678140

**Published:** 2021-05-03

**Authors:** Honghao Gao, Ying Li, Zijian Zhang, Wenbing Zhao

**Affiliations:** ^1^School of Computer Engineering and Science, Shanghai University, Shanghai, China; ^2^School of Computer, Zhejiang University, Hangzhou, China; ^3^The School of Computer Science, The University of Auckland, Auckland, New Zealand; ^4^College of Engineering, Cleveland State University, Cleveland, OH, United States

**Keywords:** machine learning, biomedical computing, intelligence, healthcare, precision medicine

In recent years, the development of biomedical imaging techniques, integrative sensors, and artificial intelligence has brought many benefits to the protection of health. We can collect, measure, and analyze vast volumes of health-related data using the technologies of computing and networking, leading to tremendous opportunities for the health and biomedical community. Biomedical intelligence, especially precision medicine, is considered one of the most promising directions for healthcare development. Meanwhile, these technologies have also brought new challenges and issues.

This Research Topic was supported by Frontiers and includes three collaborating journals: Frontiers in Genetics, Frontiers in Public Health, and Frontiers in Computer Science. We accepted 10 papers from 21 open submissions. The summaries of these papers are outlined below.

In the article entitled “Development, Validation and Comparison of Artificial Neural Network Models and Logistic Regression Models Predicting Survival of Unresectable Pancreatic Cancer” by Tong et al. the authors developed Artificial Neural Network (ANN) models based on 3, 7, and 32 basic features, predicting the survival of unresectable pancreatic cancer patients over 8 months. These models might help to optimize personalized patient management.

In the article entitled “*P*-Wave Area Predicts New Onset Atrial Fibrillation in Mitral Stenosis: A Machine Learning Approach” by Tse et al. the authors studied Chinese patients diagnosed with mitral stenosis in sinus rhythm at baseline between November 2009 and October 2016. They concluded that atrial electrophysiological alterations in mitral stenosis could be detected using electrocardiograms and based on age, and systolic blood pressure and the *P*-wave area in V3 could predict new onset atrial fibrillation (AF). They proposed a decision tree learning model, which significantly improves outcome prediction.

In the article entitled “Detection and Severity Assessment of Peripheral Occlusive Artery Disease via Deep Learning Analysis of Arterial Pulse Waveforms: Proof-of-Concept and Potential Challenges” by Kim et al. the authors demonstrated the deep learning-based arterial pulse waveform analysis contributes to the PAD screening, and they presented challenges that must be addressed for real-world clinical applications.

In the article entitled “Develop and Evaluate a New and Effective Approach for Predicting Dyslipidemia in Steel Workers” by Wu et al. the authors collected the physical examination information of thousands of steelworkers and screened out the risk factors of dyslipidemia in steelworkers. Then, based on the data characteristics, they employed the convolutional neural network to predict the risk of dyslipidemia in steelworkers.

In the article entitled “Automated Detection of Acute Lymphoblastic Leukemia From Microscopic Images Based on Human Visual Perception” by Bodzas et al. the authors proposed a novel approach based on conventional digital image processing techniques and machine learning algorithms. The traditional machine learning classifiers, the artificial neural network and the support vector machine, were used to automatically identify acute lymphoblastic leukemia from peripheral blood smear images.

In the article entitled “Review on the Application of Machine Learning Algorithms in the Sequence Data Mining of DNA” by Yang et al. the authors introduced the development process of sequencing technology. They analyzed the basic process of data mining, summary several major machine learning algorithms, and pointed out the challenges faced by machine learning algorithms in the mining of biological sequence data and possible solutions. They also reviewed four typical applications of machine learning in Deoxyribonucleic acid (DNA) sequence data.

In the article entitled “Parkinson's Disease in Teneurin Transmembrane Protein 4 (TENM4) Mutation Carriers” by Pu et al. the authors investigated clinical and genetic manifestations in four unrelated pedigrees with both essential tremor (ET) and Parkinson's disease (PD) in which TENM4 variants were identified. They discussed whether TENM4 variants contributed to the risk of developing PD. Thus, the frequency of TENM4 variants was evaluated from four PD pedigrees and other 407 subjects.

In the article entitled “Deep Learning in Head and Neck Tumor Multiomics Diagnosis and Analysis: Review of the Literature” by Wang and Li the authors reviewed the multiomics image analysis of head and neck tumors using convolution neural network (CNN) and other Deep learning (DL) neural networks. They evaluated its application in early tumor detection, classification, prognosis/metastasis prediction, and the signing out of the reports.

In the article entitled “Alzheimer's Disease Classification with a Cascade Neural Network” by You et al. the authors proposed a cascade neural network with two steps to achieve a faster and more accurate Alzheimer's Disease (AD) classification by exploiting gait and electroencephalogram (EEG) data simultaneously. They collected gait and EEG data from 35 cognitively healthy controls, 35 mild cognitive impairment (MCI), and 17 AD patients to demonstrate their proposed method.

In the article entitled “Application of Structural and Functional Connectome Mismatch for Classification and Individualized Therapy in Alzheimer Disease” by Ren et al. the authors performed a preliminary exploration into a set of Alzheimer disease data to improve the personalized approach in order to understand individual connectomes in an actionable manner. They found that there were consistent patterns of white matter fiber loss, mainly the Default Mode Network (DMN) and Deep Subcortical Structures (DSS), which were present in nearly all patients with clinical Alzheimer's disease.

In conclusion, we would like to thank all the authors who submitted their research articles to our Research Topic. We highly appreciate the contributions of the reviewers for their constructive comments and suggestions. We also would like to acknowledge the guidance from the Editor-in-Chief and staff members of Frontiers.

## Author Contributions

All authors listed have made a substantial, direct and intellectual contribution to the work, and approved it for publication.

## Conflict of Interest

The authors declare that the research was conducted in the absence of any commercial or financial relationships that could be construed as a potential conflict of interest.

